# Child and Adolescent Suicide in the Broader Area of Athens, Greece: A 13-Year Retrospective Forensic Case-Series Analysis

**DOI:** 10.3390/pediatric17040072

**Published:** 2025-07-01

**Authors:** Kallirroi Fragkou, Maria Alexandri, Konstantinos Dimitriou, Athina Tatsioni, Flora Bacopoulou, Panagiotis Ferentinos, Laurent Martrille, Stavroula Papadodima

**Affiliations:** 1 Department of Forensic Medicine and Toxicology, School of Medicine, National and Kapodistrian University of Athens, 11527 Athens, Greece; roifragou@gmail.com (K.F.); malexandri@gmail.com (M.A.); konstantine.dimitriou@gmail.com (K.D.); 2 Research Unit for General Medicine and Primary Health Care, Faculty of Medicine, School of Health Sciences, University of Ioannina, 45110 Ioannina, Greece; atatsion@uoi.gr; 3 Center for Adolescent Medicine and UNESCO Chair in Adolescence Health Care, First Department of Pediatrics, School of Medicine, National and Kapodistrian University of Athens, Aghia Sophia Children’s Hospital, 11527 Athens, Greece; fbacopoulou@med.uoa.gr; 4 Affective Disorders and Suicide Unit, 2nd Department of Psychiatry, “Attikon” University General Hospital, School of Medicine, National and Kapodistrian University of Athens, 11527 Athens, Greece; pferentinos@med.uoa.gr; 5 Department of Legal Medicine CHU Montpellier, University of Montpellier, 34000 Montpellier, France; laurent.martrille@chu-montpellier.fr

**Keywords:** suicides, children, adolescents, pediatric, autopsy, forensic, cause of death, psychiatric, toxicological, suicide note

## Abstract

Purpose: Suicide is a leading cause of death among children and adolescents worldwide. This study examined the prevalence and characteristics of suicides among children and adolescents (aged ≤ 19 years) over a 13-year period in the broader area of Athens, Greece. Key aspects analyzed included victim demographics, circumstances surrounding the incidents, and methods employed. Methods: A retrospective analysis was conducted on autopsy cases performed at the Department of Forensic Medicine and Toxicology, National and Kapodistrian University of Athens, from 1 January 2011, to 31 December 2023. Results: Out of 5819 autopsies conducted between 2011 and 2023, 371 were classified as suicides. Among these, 12 cases (representing 3.2% of suicides) involved children and adolescents aged ≤ 19 years and met the study’s inclusion criteria for detailed forensic analysis. The average age of the victims was 17.7 ± 2.1 years (range: 14–19), with males representing 58.3% of cases. Hanging was the most common method of suicide (9 cases, 75.0%), followed by firearm use, falls from height, and hydrogen sulfide inhalation (one case each). Death occurred in the home in 10 cases (83.3%), with 6 specifically taking place in the bedroom. Scars indicative of prior self-harming behavior were present in two cases (16.7%), while suicide notes were found in three cases (25.0%). Toxicological analysis revealed alcohol and cannabis use in one case, cannabis alone in one case, and alcohol alone in two cases. Four victims (33.3%) had a documented psychiatric diagnosis, with two of them under antidepressant treatment at the time of death. Conclusions: This study highlights the forensic value of autopsy-based investigations in unveiling hidden patterns of adolescent suicidality and informs targeted prevention strategies. Integrating medico-legal findings into public health responses may enhance early identification and intervention in vulnerable youth populations.

## 1. Introduction

Suicide remains a leading cause of death among children and adolescents worldwide, reflecting a complex interplay of psychological, social, and environmental factors [1,2,3]. Adolescents, in particular, face unique vulnerabilities, including developmental changes, social pressures, and mental health challenges, which can contribute to suicidal ideation and behavior [4].

According to UNICEF, suicide is the second leading cause of death among individuals aged 15–19 years in Europe, with approximately 931 youth suicides reported across the EU in 2020—equivalent to 18 deaths per week. Males account for nearly 70% of these deaths [5]. Suicide rates vary across Europe, with Lithuania recording approximately 14 suicides per 100,000 adolescents, Estonia 11 per 100,000, and Norway, Finland, and Ireland around 8 per 100,000 [6].

In contrast, suicide rates among children under the age of 15 years are significantly lower than those among adolescents. The World Health Organization’s Global Health Estimates reported 105,684 suicides among individuals aged 15–29 years in 2021, compared to 5974 suicides among children aged 5–14 years [7].

Greece consistently ranks among the European countries with the lowest suicide rates. Over the decade 2000–2009, the suicide rate among individuals aged 10–24 years in Greece showed a declining trend, averaging 1.3 per 100,000 annually and accounting for 4.8% of total external-cause mortality in this age group. Most suicides occurred in young adults (64%), followed by older adolescents (31%) [8]. In 2020, while the overall EU suicide rate stood at approximately 10.2 per 100,000, Greece, Cyprus, and Malta reported substantially lower rates ranging between 3.5 and 4.0 per 100,000 [9]. More recent estimates indicate that the suicide rate in Greece is as low as 0.18 per 100,000 among individuals aged 10–14 years [10].

The above epidemiological figures, drawn from diverse sources and based on differing age classifications, are not directly comparable; however, they illustrate broader trends across Europe and Greece and serve to contextualize the present study.

These low rates may be partially attributed to protective geographical and sociocultural factors, including favorable climate, short distances between communities, strong family ties, and the preservation of traditional lifestyles [11,12]. Nevertheless, modern stressors such as urbanization and the increasing influence of digital platforms introduce new risks, including cyberbullying and online access to information on lethal methods [12].

Despite these developments, forensic research focusing specifically on youth suicides in Greece remains limited. Existing studies have largely centered on adult populations or general suicide trends [13,14,15,16]. According to the Hellenic Statistical Authority, between 2011 and 2022, 477 suicides were documented among individuals aged 10–14 years and 1508 among those aged 15–19 years. No suicide-related deaths were recorded among younger children [17]. However, these national registry data provide only basic demographic and mortality information and lack the detailed psychological, toxicological, and contextual insights that forensic autopsies can uniquely offer.

The forensic perspective is increasingly recognized as vital in deepening the understanding of youth suicide. Systematic reviews emphasize the importance of autopsy and psychological autopsy data in identifying risk factors and clarifying circumstances surrounding child and adolescent suicides. Soole et al. (2015) highlighted that many child suicides occur without a prior psychiatric diagnosis and are often precipitated by acute familial conflicts, with hanging being the most prevalent method [18]. In a 10-year forensic review from Canada, Shaw et al. (2005) found that 87% of youth suicides involved adolescents aged 15–19 and were often associated with psychological stressors, substance use, and depressive symptoms [19]. More recently, Pacchioni et al. (2023) reaffirmed mental illness—particularly mood, substance use, and personality disorders—as primary risk factors, while stressing the methodological variability across forensic studies and the urgent need for standardization [20].

The objective of this study is to present a 13-year retrospective forensic case series of child and adolescent suicides in the broader area of Athens, Greece. Rather than aiming to generalize incidence rates, this study seeks to offer in-depth insight into the specific forensic patterns of these rare but tragic deaths by synthesizing autopsy results, toxicological analyses, family histories, and police investigation findings. Where available, psychiatric and psychosocial information is also considered to enhance contextual understanding. Ultimately, it aims to inform public health strategies and contribute to the international discourse on youth suicide through the lens of forensic medicine.

## 2. Materials and Methods

### 2.1. Study Design

This is a retrospective forensic case series study based on autopsy records spanning a 13-year period, from 1 January 2011, to 31 December 2023. The analysis was conducted at the Department of Forensic Medicine and Toxicology, National and Kapodistrian University of Athens. The study was approved by the University’s Ethics Committee as part of a broader research project on causes of death in individuals with a psychiatric history (Approval No. 631/05-05-2022). All data were processed anonymously to preserve confidentiality.

### 2.2. Sample Selection

The source population comprised 5819 consecutive forensic autopsies conducted during the study period. Cases were eligible for inclusion if they met the following criteria:

- The deceased was aged 19 years or younger
- The manner of death was certified as suicide
- The case was referred by investigative authorities due to its classification as sudden, unexpected, or violent death
- A complete forensic autopsy had been conducted, including both external and internal examination and toxicological examination

All included cases were examined by the same forensic pathologist (SP), ensuring consistency in the approach to documentation and interpretation.

### 2.3. Data Collection Procedures

Retrospective case review was performed in 2024 by two physicians with three years of postgraduate training in forensic medicine (KF and MA), in collaboration with a board-certified forensic pathologist (KD). A standardized data extraction sheet was used to collect the following information for each case:

- Demographic data (age and gender)
- Autopsy findings: (a) cause of death, (b) external examination: recent injuries, scars indicating prior self-harming behavior, tattoos with a special meaning, (c) findings from internal examination if abnormal.
- Toxicological analysis results
- Medical and psychiatric history, where available
- Information provided by next of kin (e.g., family dynamics, recent stressors)
- Police investigation findings (scene characteristics, suicide notes, digital evidence)

Information obtained from next of kin was reviewed for evidence of recent stressors, family dynamics, behavioral changes, school or peer-related issues, and psychiatric history. Similarly, police investigation reports were examined for scene characteristics, method of suicide, presence of suicide notes, digital footprints (e.g., internet searches, messages), and any indication of third-party involvement. To distinguish significant from non-significant information, a relevance-based approach was used. Information was considered significant if it had a direct bearing on the victim’s mental state, suicide planning, or potential triggers. This assessment was performed jointly by two physicians under training in forensic medicine and a senior forensic pathologist, with disagreements resolved through consensus.

## 3. Results

A total of 5819 autopsies were reviewed during the 13-year study period, among which 371 were classified as suicides. Twelve of these involved individuals aged 19 years or younger, meeting the study’s inclusion criteria (See Table 1).

The victims had an average age of 17.7 ± 2.1 years (range: 14–19), and the majority were male (7 cases, 58.3%). Hanging was the predominant method of suicide (9 cases, 75.0%). The remaining methods included the use of a shotgun (1 case), falling from a height (1 case), and intentional inhalation of hydrogen sulfide gas, which had been produced using instructions found online (1 case).

No defensive wounds or other indicators of third-party involvement were identified in any case. In all cases of hanging, the victims were found with the ligature still present at the scene (e.g., belts, scarves, bathrobe cords), consistent with self-suspension. Autopsy confirmed typical forensic findings such as ligature marks compatible with hanging and conjunctival petechiae and the absence of defensive injuries or signs of struggle.

Most suicides occurred in private settings: 10 of the 12 deaths (83.3%) took place in the victim’s home, with 6 specifically occurring in bedrooms.

Indicators of psychological vulnerability were identified across several cases. Four victims (33.3%) had an established psychiatric diagnosis, and in two of these, therapeutic levels of antidepressants were confirmed through toxicological analysis. However, signs of psychological distress were also present in individuals without formal diagnoses. These included prior self-harm (2 cases, 16.7%), reported experiences of bullying and physical assault (1 case), difficulties adjusting after a recent move (1 case), and observations by family of emotional disturbance or social withdrawal.

Suicide notes were found in three cases (25.0%). Their contents varied; one included a warning about hazardous gas exposure, another was addressed to a sibling and reflected personal thoughts, while the third was accompanied by psychotropic medication and alcohol, suggestive of significant emotional turmoil.

Toxicological testing revealed substance use in four cases (33.3%). Alcohol was present in three, and cannabis in two; one case involved both substances. In the firearm-related suicide, blood alcohol was detected, but not in the vitreous humor—raising the possibility of postmortem ethanol production rather than ante-mortem consumption.

Case 1: A 16-year-old female was found by her parents hanged from a tree in her home’s yard. Her parents last saw her in bed that morning and discovered her fully suspended upon returning from the supermarket. She had a known history of depression but was not receiving treatment. On external examination a ligature furrow characteristic of hanging was observed on the neck. No additional traumatic injuries were identified, and internal examination did not reveal any remarkable organ pathology. Toxicology tests were negative for any substances.

Case 2: A 19-year-old female was found by her mother hanged with a belt in her bedroom. The suspension was incomplete, with the victim’s feet in contact with the ground. No relevant psychiatric history was documented. On external examination, a ligature furrow characteristic of hanging was observed on the neck. No additional traumatic injuries were identified, and internal examination did not reveal any remarkable organ pathology. Toxicology tests were negative.

Case 3: A 14-year-old female was found inside her bedroom closet, where a strong odor of gas was present. She had produced hydrogen sulfide for inhalation, leaving a note at the entrance warning against entering without precautions. Her father reported difficulty adjusting to a recent relocation and school change. She spent long hours on Facebook. External examination revealed self-inflicted scars on the forearms and right hip. The forearm scars appeared older and healed, while the red scars on the right hip were more recent, estimated to be 1–2 months old at the time of death. Autopsy revealed the presence of gastric contents in the lungs, indicative of gastric aspiration. Histological examination of the heart showed myocardial atrophy, suggestive of possible nutritional deficiencies. No additional pathological findings were observed in the remaining internal organs. Toxicology tests revealed 6.8% carboxyhemoglobin.

Case 4: A 19-year-old male was found by his mother hanged inside a sheepfold. A belt with a metal buckle and hook was used. No prior suicide attempts, notes, or psychiatric history were reported. On external examination a ligature furrow characteristic of hanging was observed on the neck. No additional traumatic injuries were identified, and internal examination did not reveal any remarkable organ pathology. Toxicology tests were negative.

Case 5: A 19-year-old male was found by his mother hanged in his room. A suicide note addressed to his brother was found. His brother described him as anxious and sensitive, but also egotistical, noting that he had psychological issues but was not taking medication. He was an informatics student, but he did not regularly attend his lessons. The night before his death, he was involved in a minor car accident, did not sleep, and consumed large amounts of alcohol and tobacco. On external examination a ligature furrow characteristic of hanging was observed on the neck. No additional traumatic injuries were identified, and internal examination did not reveal any remarkable organ pathology. Toxicology tests revealed 0.75 g/L ethanol in his blood and THC metabolites in his urine.

Case 6: A 19-year-old male was found hanged in the yard. His mother had died when he was 10, which he had witnessed. He was the only child in the family. He had been diagnosed with depression and was under psychiatric care. On the day of the incident, he canceled his appointment but spoke to his psychiatrist on the phone. On external examination a ligature furrow characteristic of hanging was observed on the neck. No additional traumatic injuries were identified, and internal examination did not reveal any remarkable organ pathology. Toxicology tests revealed therapeutic levels of citalopram.

Case 7: A 16-year-old female was found by her mother lying on the floor in her bedroom, after hanging herself from the closet handle using a scarf and a chair. She was described as an excellent student with no apparent distress. On her left forearm, she had ink-written messages, including a name with the symbol of a heart (❤️) and a date, suggesting a romantic connection. No suicide note was found. On external examination a ligature furrow characteristic of hanging was observed on the neck. No additional traumatic injuries were identified, and internal examination did not reveal any remarkable organ pathology. Toxicology tests were negative.

Case 8: An 18-year-old male was found hanged with a bathrobe belt in his bedroom. He had a history of psychological issues and was on medication (Dumyrox 10 mg and Zoxil 5 mg). A suicide note was discovered. On external examination a ligature furrow characteristic of hanging was observed on the neck. No additional traumatic injuries were identified, and internal examination did not reveal any remarkable organ pathology. Toxicology analysis detected fluvoxamine and olanzapine (therapeutic levels) and 0.22 g/L ethanol.

Case 9: A 14-year-old male was found hanged in his home’s yard using a swing rope. No prior psychological issues were reported by family members. He was probably a victim of bulling, and he was assaulted two weeks ago, which resulted in an injury on his lips. The night before his death, he spent time partying with some girls and boys. On external examination a ligature furrow characteristic of hanging was observed on the neck. No additional traumatic injuries were identified, and internal examination did not reveal any remarkable organ pathology. Toxicology tests were negative.

Case 10: A 19-year-old male fell from the second floor of a house (a friend’s home) after smoking cannabis, according to witnesses. His family was unaware of any substance use and described him as happy with no depressive signs. After his death, his mother discovered phone messages referring to “haze.” Witnesses stated that he had experienced hallucinations after cannabis use and, as he calmed down, jumped, believing that “the meaning of life was death.” Autopsy revealed multiple injuries distributed across the head, thorax, abdomen, and extremities consistent with trauma sustained from the fall. Toxicology analysis confirmed the presence of Δ9-tetrahydrocannabinol and its metabolites.

Case 11: A 19-year-old female was found hanged by her grandfather with a cord from a chandelier in her bedroom. Her grandfather cut the rope. On her bedside table, an open beer can, and a glass of wine were found. External examination revealed self-inflicted scars (2–4 cm) on her left forearm. Internal examination did not reveal any remarkable organ pathology. Toxicology tests detected 0.60 g/L ethanol in her blood and in the vitreous humor.

Case 12: A 19-year-old male was found by his brother dead from a gunshot wound to the right chest, inflicted with a pump-action shotgun registered to his father. His brother discovered him on the couch of his house in the living room. His family stated that he did not have experience handling firearms. Extensive injuries were observed in the thoracic ribs, lungs, liver, and heart, resulting from the gunshot wound. The characteristics of the trauma were consistent with a close-range discharge. Toxicology tests detected 0.73 g/L ethanol in his blood, though ethanol was absent in the vitreous humor, suggesting postmortem microbial production.

## 4. Discussion

This forensic case-series analysis examines adolescent suicides in the greater Athens area over a 13-year period, with emphasis on autopsy-confirmed methods of death, toxicological findings, and psychiatric documentation. The discussion interprets these findings within the relevant forensic and psychiatric literature.

Suicide is among the leading causes of death in adolescents globally, with rates rising significantly in recent years [21,22]. While such trends are well-documented internationally, national-level data from Greece remain limited. Our study contributes forensic insight into local patterns, revealing specific constellations of risk factors—including psychiatric morbidity, substance use, and signs of emotional distress—across the examined cases.

The adolescent period entails neurodevelopmental changes, emotional volatility, and social role formation, all of which may contribute to suicidal vulnerability [23,24,25]. When such vulnerabilities are reflected in forensic findings—such as self-inflicted injuries, toxic substance exposure, or method selection—they gain diagnostic weight and underscore the importance of comprehensive postmortem investigation.

### 4.1. Methods of Suicide

In our case series, hanging was the most frequently observed method of suicide, occurring in 9 out of 12 cases (75.0%), a pattern that has also been reported in previous studies from Greece [8,11] and other European countries [26,27]. Autopsy examination confirmed typical features of suicidal hanging, including ligature furrows, suspension point positioning, and absence of defensive injuries. In one case, the victim utilized a closet handle for partial suspension, illustrating the potential for suicide with minimal vertical drop—an important forensic point when evaluating scene plausibility [28]. The accessibility and lethality of hanging contribute to its prevalence, particularly in domestic settings where materials such as belts, ropes, and scarves are readily available [29].

Other methods observed in our study included firearm use (one case), falls from height (one case), and poisoning (one case). The firearm-related suicide was an isolated case, reflecting Greece’s strict gun control laws, which limit access compared to countries such as the United States, where firearms are a leading method among adolescents [30]. Jumping from heights, another common method in youth suicides, was also observed in our study, consistent with previous findings that indicate an overrepresentation of this method in younger populations [26].

Poisoning remains a significant concern, particularly among female adolescents, who are more likely to engage in this method [31]. In our study, a 14-year-old girl used hydrogen sulfide gas after obtaining instructions online, underscoring the growing role of digital platforms in influencing suicide methods [32]. The dissemination of dangerous content through the internet presents new challenges in suicide prevention, emphasizing the need for enhanced monitoring and intervention strategies to mitigate the impact of harmful online materials.

Overall, our findings reinforce the importance of restricting access to highly lethal methods, implementing targeted prevention strategies, and increasing awareness about emerging risks, such as the influence of the internet on suicide behaviors. Future research should further explore the sociocultural factors that shape method selection and identify effective interventions to reduce youth suicide rates.

### 4.2. Psychiatric Disorders and Psychological Distress

Mental disorders are established risk factors for adolescent suicide, particularly mood, anxiety, and trauma-related conditions [33,34,35]. However, the relationship between psychiatric morbidity and suicidal behavior is complex and not uniformly present. In our case series, only 4 of the 12 individuals (33.3%) had a documented psychiatric diagnosis prior to death. Among these, two were receiving pharmacological treatment, with therapeutic drug levels confirmed postmortem—providing forensic support for treatment adherence. Although the presence of psychiatric diagnoses was documented in four cases, the specific diagnostic categories (e.g., major depressive disorder, anxiety disorders, or personality disorders) were not consistently available in the records reviewed. This lack of diagnostic specificity reflects limitations in the medical documentation provided to the forensic team, which often did not include full psychiatric histories or structured assessments.

Despite the low rate of formal diagnosis, indirect evidence of psychological distress was present in several cases. Case 3 exhibited both self-harming behavior and difficulty adjusting to recent life events; Case 5 presented with substance use and emotional instability in the context of academic failure and social strain; Case 9 had a history of bullying and physical assault; and Case 10 experienced perceptual disturbances following cannabis use. Such findings highlight the importance of recognizing subclinical or undiagnosed psychiatric burden during forensic investigation.

The discrepancy between observable distress and formal diagnosis aligns with prior research indicating that up to 60% of youth suicide victims may lack a documented mental health condition [36]. Factors such as atypical symptom presentation, stigma, and limited access to care contribute to underdiagnosis, particularly in adolescents with co-occurring conditions such as ADHD or autism spectrum disorders [1,37,38,39,40,41].

From a forensic perspective, these observations underscore the critical role of autopsy, scene investigation, and collateral history in identifying psychosocial vulnerabilities. Self-inflicted injuries, behavioral patterns, and contextual clues may serve as valuable markers of unrecognized psychiatric risk, particularly when formal medical records are sparse.

### 4.3. Suicide Notes

In our case series, suicide notes were recovered in 3 of the 12 cases (25.0%). The observed frequency in our study is consistent with prior European findings, where the prevalence of suicide notes among all age groups ranged between 25.0% and 36.7% in [14,42]. Freuchen and Grøholt reported even higher rates (55%) among children and adolescents under 15 in Norway, with notes reflecting perceived burdensomeness and emotional distress [43]. Our findings support the view that, even in young individuals, suicide is not always an impulsive act and may involve deliberate reflection.

From a forensic perspective, the presence of a suicide note adds contextual depth to the manner-of-death determination but should be evaluated alongside other investigative and autopsy findings. The absence of a note does not exclude intentionality, especially in adolescent suicides, where impulsivity, emotional fluctuation, and privacy concerns may influence the decision to leave a final message [43].

### 4.4. Previous Suicide Attempts and Self-Harm

Prior suicide attempts are among the most significant risk factors for completed suicide in adolescents. Ruch et al. in their study examining characteristics and precipitating circumstances of suicide among children aged 5 to 11 years in the United States during the period 2013–2017, found that 11.9% of child victims had a reported history of suicide attempts, 23.7% had experienced suicidal ideation, and 25.4% had previously made suicidal statements or expressed death wishes before their suicide [44]. In our case series, scars indicative of prior self-harming behavior were observed in 2 of the 12 individuals (16.7%), which may reflect previous suicide attempts or non-suicidal self-injury, aligning with findings that emphasize the heightened risk associated with unresolved underlying issues [45].

Self-harm behaviors, such as cutting, are also critical indicators of prolonged psychological distress in adolescents. These behaviors often precede suicidal actions, providing opportunities for early intervention. Addressing self-harm through counseling, support networks, and targeted mental health services can significantly mitigate the risk of escalation to suicide [46].

### 4.5. Demographics: Age and Gender Trends

Our study identified a slight male predominance (7 out of 12 cases, 58.3%) among child and adolescent suicide victims, aligning with global trends [47,48]. Research consistently shows that males are more likely to complete suicide, whereas females are more prone to non-lethal attempts and self-harming behaviors. A study by Rodway et al. in the United Kingdom found that between 2014 and 2016, 71% of suicides among young people aged 10–19 years occurred in males, reinforcing this gender disparity [49].

Females tend to exhibit more warning signs, such as verbal threats, prior suicide attempts, and non-suicidal self-injury (NSSI), offering greater opportunities for intervention [50,51].

The average age of victims in our study was 17.7 years, underscoring late adolescence as a critical period of vulnerability for suicidal behavior [25]. Notably, no cases of suicide were recorded among children younger than 10 years, consistent with findings that suicide is rare in early childhood but increases significantly during adolescence.

### 4.6. Substance Use and Toxicological Findings

Substance use is a critical factor in adolescent suicides, with toxicological analyses frequently revealing the presence of alcohol, cannabis, and prescription medications. In this series, toxicology played a key role in contextualizing the victims’ mental states and potential impulsivity. Substances were identified in 4 of the 12 cases, most commonly alcohol and cannabis. Blood ethanol concentrations ranged from 0.22 to 0.75 g/L. In one firearm-related death (Case 12), ethanol was detected in peripheral blood but absent from the vitreous humor, suggesting postmortem microbial production rather than ante-mortem consumption.

Pharmacological toxicology also provided valuable insight into treatment adherence. In Cases 6 and 8, the presence of antidepressants (citalopram and fluvoxamine, respectively) at therapeutic levels confirmed compliance with prescribed psychiatric medications. This indicates that half of the victims with known psychiatric diagnoses were actively receiving pharmacological treatment at the time of death.

Adolescents who misuse substances face a significantly higher risk of suicidal ideation and attempts, as substance use can exacerbate underlying psychiatric disorders, heighten impulsivity, and impair judgment, ultimately contributing to suicidal behaviors. A study by Yang et al. based on data from the Youth Risk Behavior Survey, found that adolescents engaging in polysubstance use were three to five times more likely to experience suicidal thoughts, make a suicide plan, or attempt suicide compared to non-users [52]. Similarly, Jones et al. reported that adolescents with substance use disorders were approximately four times more likely to consider suicide and five times more likely to plan or attempt suicide than those without such disorders [53].

Among substances, alcohol is a particularly influential factor. Research suggests that adolescents who engage in heavy drinking are up to 17 times more likely to die by suicide compared to their non-drinking peers [54]. Cannabis use has also been strongly associated with an increased risk of depression and suicidality, with longitudinal studies indicating a higher incidence of suicide attempts among frequent users [55]. Prescription medications, particularly antidepressants and antipsychotics, play a complex role in adolescent mental health. While essential for managing psychiatric disorders, some medications—especially selective serotonin reuptake inhibitors (SSRIs)—have been linked to an increased risk of suicidal thoughts in younger populations [56].

Postmortem toxicological analyses highlight the frequent co-occurrence of multiple substances, which may amplify their harmful effects. Polysubstance use is common among adolescent suicide victims, with combinations such as alcohol and cannabis or alcohol and psychotropic medications increasing the likelihood of impulsive, fatal outcomes [57].

In our study, polysubstance use was evident in Case 5, where both ethanol (0.75 g/L) and THC metabolites were present. This combination is known to impair judgment, heighten impulsivity, and reduce inhibition [58]. The context—a minor car accident, emotional distress, and subsequent hanging—demonstrates how forensic toxicology can uncover contributing factors that may not be evident through history alone. In Case 8, fluvoxamine, olanzapine, and ethanol were detected. Although drug levels were within therapeutic ranges, the co-ingestion of alcohol may have contributed to emotional destabilization [59,60]. Importantly, the interpretation was not one of overdose or poisoning but of a possible synergistic effect facilitating suicidal behavior. This underscores the necessity of evaluating toxicology results not solely by concentration but also in relation to psychiatric history and behavioral context.

### 4.7. Location of Suicides

In our case series, 10 of the 12 deaths (83.3%) occurred in the victim’s home, with 6 of these taking place specifically in the bedroom. While these observations may reflect environmental familiarity and privacy, they should be interpreted cautiously due to the small sample size.

This finding aligns with prior research indicating that the home environment is the most frequent setting for self-inflicted deaths among children and adolescents. Studies analyzing child suicides have shown that an overwhelming majority take place at home, particularly in the child’s bedroom. For example, Sheftall et al. [34] reported that 95.6% and 85.8% of suicide cases among children aged 5 to 11 years and early adolescents aged 12–14 years, respectively, occurred in the home environment, with hanging being the predominant method. Similarly, Ruch et al. [44] found that most child suicide deaths occurred in the family home (95.5%), with 65.6% specifically in the child’s bedroom, highlighting the critical role of environmental accessibility in impulsive suicidal behaviors.

Given the above observations, suicide prevention strategies may benefit from addressing environmental risk factors within domestic spaces. The accessibility of ligature points, medications, or firearms may contribute to increased risk in moments of acute distress. The American Academy of Child and Adolescent Psychiatry (AACAP, 2024) [61] recommends that firearms be stored unloaded in a locked safe, with ammunition stored separately, while sharp objects such as knives and razor blades should be kept securely out of reach.

### 4.8. Limitations of the Study

Despite its strengths in detailed forensic documentation, this study has several limitations. First, the number of child and adolescent suicide cases (n = 12) was small, also reflecting the rarity of completed suicide in this age group in Greece. This limits statistical power and generalizability.

Second, the retrospective nature of the study means that psychosocial variables were often underreported or absent, particularly in cases lacking formal psychiatric diagnoses. Forensic documentation is primarily focused on immediate medical, anatomical, and toxicological findings; deeper psychiatric or psychological histories may not be available unless provided by family members or investigative authorities. This restricts the ability to explore nuanced psychiatric dynamics in detail.

Third, psychiatric diagnoses relied on medical records or family reporting, without access to structured psychological assessments or therapeutic records. As such, undiagnosed or misdiagnosed conditions could not be accounted for, leading to possible underestimation of mental health morbidity.

Lastly, the study is geographically restricted to the broader Athens area and cannot be extrapolated to rural or island regions, where sociocultural dynamics and access to mental health services differ.

Nonetheless, the strength of this study lies in its autopsy-based foundation, offering a rare forensic lens into youth suicide and supplementing a scarce national dataset with case-level insights.

### 4.9. Implications for Prevention

Suicide prevention among children and adolescents requires a multifaceted approach that addresses risk factors at individual, familial, and societal levels. Although most cases in our study lacked documented psychiatric diagnoses or overt warning signs, it is likely that subtle indicators were missed. Enhanced awareness and education among caregivers, educators, and clinicians could facilitate earlier detection of distress and prevent escalation. Strengthening social connectedness within families and communities is a crucial protective factor, as it helps reduce feelings of isolation and emotional distress. Research indicates that strong social support networks significantly lower the risk of lifetime suicide attempts [62].

In the context of primary healthcare, screening for anxiety disorders, depression, and suicidal ideation in adolescents (ages 12–18) is recommended for the general adolescent population, including those without symptoms [63,64]. Additionally, screening for intimate partner violence is advised for women of reproductive age, including adolescent girls [65]. A fundamental requirement for effective implementation is the integration of primary healthcare services with mental health and social services to ensure accurate diagnosis and appropriate intervention.

Restricting access to lethal means, such as firearms and toxic substances, is another essential preventive strategy. Counseling families on the safe storage of firearms and medications has been shown to be an effective measure in reducing suicide risk [66].

Addressing substance use is also a key component of suicide prevention, as substance misuse can exacerbate mental health challenges and increase impulsivity. Targeted interventions—such as education, early intervention programs, and public awareness campaigns—play a critical role in mitigating the impact of substance use on adolescent mental health [67].

In today’s digital age, promoting safe online environments is more important than ever. Exposure to harmful content, cyberbullying, and online harassment can contribute to suicidal ideation among young individuals. Collaborative efforts involving technology companies, policymakers, educators, and parents are necessary to create safer digital spaces and reduce these risks [7,68].

## 5. Conclusions

This study offers one of the few autopsy-based analyses of child and adolescent suicides in Greece, providing a detailed forensic perspective on a population group often underrepresented in the literature. Despite the small sample size, our findings reveal critical patterns—such as the predominance of home settings, the presence of self-inflicted injuries, and signs of psychosocial stress—that underscore the complex and often hidden nature of youth suicidality.

From a forensic standpoint, our results emphasize the importance of comprehensive death scene investigations, meticulous autopsy documentation, and collaboration with psychiatric and toxicological experts to identify markers of vulnerability. Suicide notes, self-harm scarring, medication levels, and behavioral changes should be integrated into a systematic approach to differentiate suicide from other manners of death in this age group.

Practically, the study supports targeted prevention strategies focusing on environmental risk factors in domestic settings and encourages the implementation of psychological autopsy protocols in cases involving minors. Integrating forensic insights into public health surveillance can help identify high-risk profiles and inform national suicide prevention frameworks. Ultimately, these efforts contribute to a more robust medico-legal response to child and adolescent suicide—one that prioritizes prevention, early identification, and intersectoral collaboration.

## Figures and Tables

**Table 1:** Suicide cases of children and adolescents.

Case No.	Age	Sex	Method	Toxicology Findings	Psychiatric History	Suicide Note	Previous Suicide Attempts or Self-Harm	Place of Death
1	16	F	Hanging	Negative	Depression (no treatment)	No	No	Her home’s yard
2	19	F	Hanging	Negative	None	No	No	Her bedroom
3	14	F	Gas inhalation	6.8% carboxyhemoglobin	None	Yes	Yes	Her bedroom
4	19	M	Hanging	Negative	None	No	No	Sheepfold
5	19	M	Hanging	0.75 g/L ethanol, THC metabolite	Psychological issues (no treatment)	Yes	No	His bedroom
6	19	M	Hanging	Therapeutic levels of citalopram	Depression (under treatment)	No	No	His home’s yard
7	16	F	Hanging	Negative	None	No	No	Her bedroom
8	18	M	Hanging	Fluvoxamine, ethanol, olanzapine	Psychological issues (under treatment)	Yes	No	His bedroom
9	14	M	Hanging	Negative	None	No	No	His home’s yard
10	19	M	Falling from height	Δ9-THC, THC metabolite	None	No	No	Friend’s home
11	19	F	Hanging	0.60 g/L ethanol	None	No	Yes	Her bedroom
12	19	M	Gunshot	0.73 g/L ethanol (possible postmortem production)	None	No	No	Living room (his home)

## Data Availability

Data is unavailable due to privacy and ethical restrictions.
